# Correction: Proceedings of the Patient Reported Outcome Measures (PROMs) Research Conference, Sheffield 2023: 22nd June 2023, University of Sheffield, Sheffield, UK

**DOI:** 10.1007/s11136-025-03980-5

**Published:** 2025-05-15

**Correction: Quality of Life Research**

10.1007/s11136-024-03887-7

The copyright holder for this article was incorrectly given as ‘© The Author(s), under exclusive licence to Springer Nature Switzerland AG 2025’ but should have been ‘© Author(s) 2025’.

The original article has been corrected.

